# Health professions students’ approaches towards practice-driven ethical dilemmas; a case-based qualitative study

**DOI:** 10.1186/s12909-023-04089-4

**Published:** 2023-05-02

**Authors:** Phyu Hnin Hlaing, Ahmed Hasswan, Vida Salmanpour, Sarra Shorbagi, Tahra AlMahmoud, Feras Jassim Jirjees, Sausan Al Kawas, Salman Yusuf Guraya, Nabil Sulaiman

**Affiliations:** 1 College of Medicine, University of Sharjah, Sharjah, United Arab Emirates; 2 College of Medicine and Health Science, United Arab Emirates University, Sharjah, United Arab Emirates; 3 College of Pharmacy, University of Sharjah, Sharjah, United Arab Emirates; 4 College of Dental Medicine, University of Sharjah, Sharjah, United Arab Emirates

**Keywords:** Medical ethics, Health professions students, Inter-professional education, Ethical dilemmas, Ethical decision

## Abstract

**Background:**

In healthcare practice, ethical challenges are inevitable and their optimal handling may potentialy improve patient care. Ethical development in medical education is critical for the transition from a medical and health sciences student to an ethical healthcare practitioner. Understanding the health professions students’ approaches towards practice-driven ethical dilemmas could harness i the effective ethical development in their medical education. This study attempts to identify the health professions students’ approaches towards practice-driven ethical dilemmas.

**Methods:**

An inductive qualitative evaluation was conducted on six recorded videos of health professions students’ case-based online group discussions, followed by a one-hour online ethics workshop. The online ethics workshop was organized with students from the College of Medicine, College of Dental Medicine and College of Pharmacy at the University of Sharjah, and the College of Medicine at the United Arab Emirates University. . The recorded videos were transcribed verbatim and imported to the qualitative data analysis software of MAXQDA 2022. Data were analyzed applying four stages of review, reflect, reduce and retrieve and two different coders triangulated the findings.

**Results:**

Six themes emerged from the qualitative analysis of the health professions students’ approaches to the practice-based ethical dilemmas; (1) emotions, (2) personal experiences, (3) law and legal system, (4) professional background, (5) knowledge of medical research and (6) inter-professional education. In addition, during the case-based group discussions in the ethics workshop, students efficiently applied the relevant ethical principles of autonomy, beneficence, non-maleficence and justice in their reasoning process to reach an ethical decision.

**Conclusion:**

The findings of this study explained how health professions students resolve ethical dilemmas in their ethical reasoning process. This work sheds light on ethical development in medical education by gaining students’ perspectives in dealing with complex clinical scenarios. The findings from this qualitative evaluation will aid academic medical institutions in developing medical and research-based ethics curriculum to transform students to ethical leaders.

## Background

In healthcare practice, ethical challenges are inevitable and optimal handling of them may lead to improve patient care. Understanding how healthcare professionals identify and responding to ethical dilemmas have been widely recognized as paramount for effective patient care. A great deal of research has been published on factors that potentially influence healthcare professionals’ decisions on ethical dilemmas. Social determinants such as age, gender, work experiences, and education are some of the contributing factors to healthcare professionals’ ethical decision making [1]. Specifically, for the community pharmacists, ‘age’ and ‘work experience’ have had a substantial impact on ethical decision-making [2], while care taker’s involvement in decision-making is an important contributing factor for home-care settings [3]. Availability, accessibility, and affordability of either patient’s attendance or care takers is some of the influencing factors to consider while trying to resolve ethical dilemmas [4]. Effective interdisciplinary communications among healthcare team members also informed ethical decisions [5]. Different health professionals may adopt a wide range of strategies for dealing with ethical dilemmas such as personal beliefs, professional training and ethical codes of conduct [6, 7].

Scholars across the world have contributed to our current understanding of ethical decision-making in clinical practice and medical research in many ways. Some researchers have focused on the theoretical models to explain the process of ethical decisions in general [8], while others have investigated specific case studies to gain insight into real-life dilemmas that clinician encounter in their practice [9]. There are several theoretical or philosophical models for developing ethical reasoning [10, 11]. The two reasoning process models that are often referred to in ethics research are casuistry and utilitarianism [12]. Studies of ethical decision-making showed the importance of the levels of moral reasoning to address the ethical dilemmas; “intuitive” and “critical” [13]. Current research recognizes the role played by practice-based models of ethical decision making that are based more on experiences than theory [14, 15]. Case-based reasoning involves an individual’s ethical decision-making, based on a specific case or set of cases and then applying lessons learned from past decisions to similar future situations. There is also broad agreement that medical ethics education should focus on real case studies rather than theory [16–18]. According to a study from Pakistan, medical ethics educators believed that using actual case scenarios was the best way to demonstrate medical students’ thought processes in elevating their approaches towards ethical practice [19].

When transitioning from a health profession student to a healthcare practitioner, ethical development in medical education is crucial. Teaching and learning medical ethics are important components of curricula in medical and health schools. While much of medical ethics education still focuses on teaching conceptual knowledge and analytical abilities, certain courses now include a special attention towards behavioral skills [20]. Several medical schools have implemented specific curricula which envisage at improving interactional skills related to ethics [21]. Medical and health professions Colleges in the United Arab Emirates (UAE) offer a wide range of medical courses including ethics throughout the whole study period.

As the role of the healthcare professional is greatly shaped by the moral conscience of the individual, teaching medical ethics at an early stage is imperative to mould the ethical attitude of future doctors. Not only in medical education, but also in nursing education, one nursing ethics study has reported that the healthcare ethics is sometimes considered to be more important than technical skills in, but ethical development has been neglected [22]. Nursing students in Iran also struggled with ethical decisions when making ethical judgements in clinical settings. Students lacked the knowledge and skills to enable them to make complex ethical choices that could have embraced consequences for their future practice [23]. Therefore, medical ethics education has become an important component of healthcare programs throughout the Middle East. However, there is a dearth of literature about medical ethics education in the UAE. 

### Teaching medical ethics at the College of Medicine (CoM), University of Sharjah (UoS)

Concerning the teaching of ethics in the CoMUoS, as one of the learning outcomes, students are expected to :apply four principles of Beauchamp and Childress (autonomy, beneficence, non-maleficence, justice)[24], analyze an incident with ethical dilemmas encountered during clinical training and apply decision-making principles to resolve ethical dilemmas.

### Teaching medical ethics in the College of Pharmacy (CoP), UoS

The CoP, UoS offers a compulsory didactic course in ethics, which focuses on epistemological (i.e., competence) and attitudinal (i.e., virtue) objectives. It includes law and ethics, and explains different ethical principles outlined in practicing pharmacy. .

### Teaching medical ethics at the College of Dental Medicine (CDM), UoS

The CDM at UoS has a competency-based 5-year Bachelor of Dental Science (BDS) curriculum with ethics and professionalism being taught as a part of the dental clinical practice in the final year.

### Teaching medical ethics in the College of Medicine and Health Sciences (CMHS), United Arab Emirates University (UAEU)

CMHS at UAEU overs a six-year medical doctor undergraduate program. This consists of two years of premedical, two preclinical courses including ethics courses and two years of clinical clerkships. The competency-based curriculum includes lectures, small group tutorials, and interactive learning opportunities.

This study attempted to identify the potential factors influencing the ethical decisions and the approaches towards practice-driven ethical dilemmas of the health professions students. 

## Methods

This study was a qualitative component of a quasi-experimental study resulting from an ethics workshop on health professions students. The qualitative component was described and reported in detail. The ethics workshop was recorded on Zoom application for qualitative data collection.

### Ethics workshop and small group discussions

**Fig. 1:**
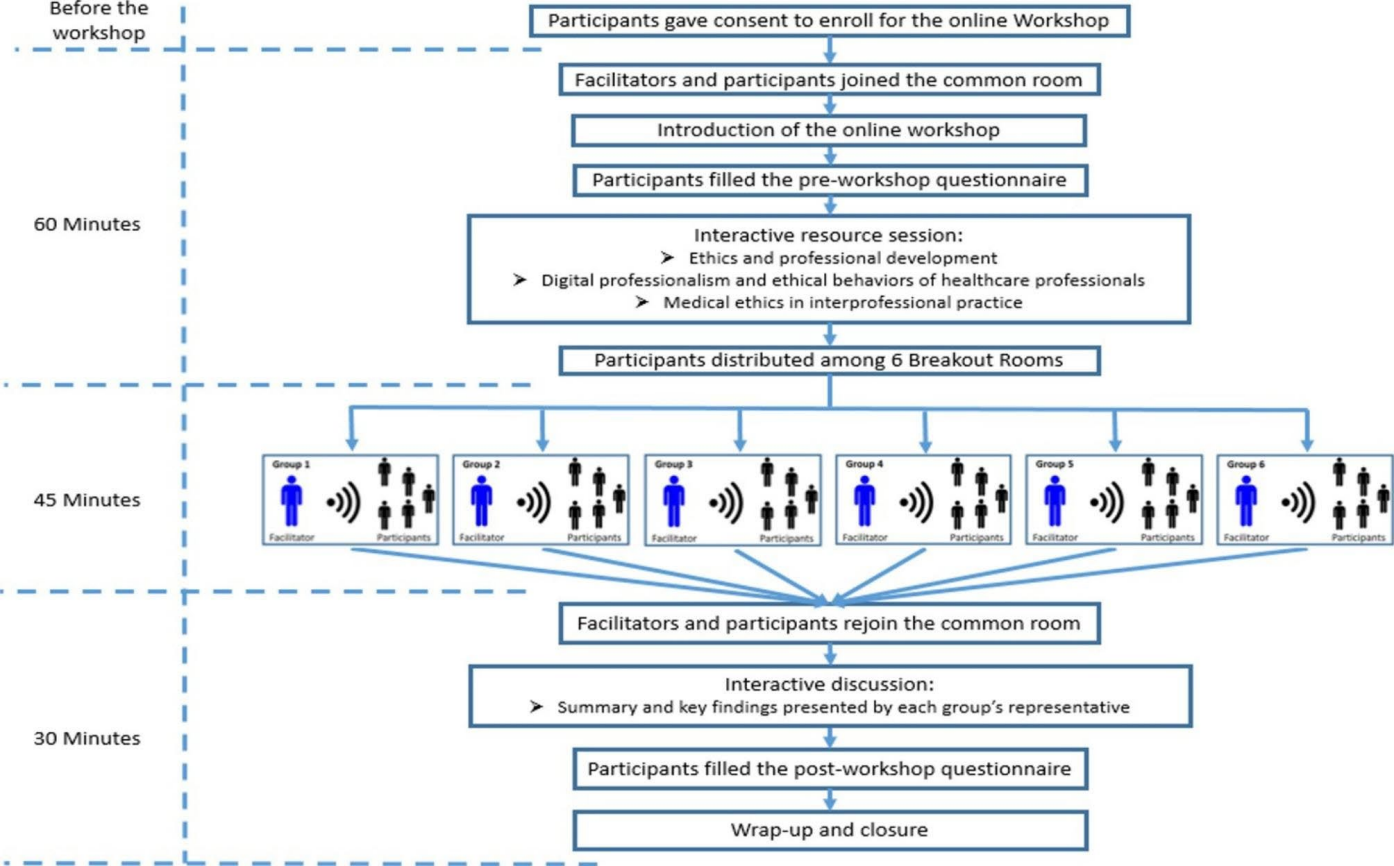
Ethics Workshop Flowchart

The ethics workshop was designed as an inter-professional learning activity, and group discussion was established between students from different colleges; medical, dental and pharmacy. The workshop lasted for two hours and consisted of a presentation and 45 min of small group discussion. The students were stratified based on their colleges and were assigned randomly to 6 small groups to discuss medical ethics scenarios. (Fig. 1).

### Participants

A case-based ethics workshop was conducted with medical, dental and pharmacy students from the CoM, CDM, and CoP at the UoS and the CMHS at the UAEU. After obtaining ethical approval from the participating institutions, student recruitment was done with involvement a person who is not a member of the research team (e.g., an administrative staff or student representative).

### Qualitative evaluation

The qualitative evaluation focused on the small group discussion sessions where students applied the ethical principles, to reach a justification for their choices. Six cases were presented by the facilitators in the breakout sessions, which included (1) a patient with mental illness refusing treatment, (2) a teenager with a suspected sexually transmitted disease, (3) good medical practice prescribing antibiotics on patient/caregiver request, (4) admitting a patient with no financial support to get treatment, (5) premarital screening-informing the partner about sexually transmitted diseases, and (6) ethics principles in medical research during COVID-19 pandemic.

An inductive approach was applied to the qualitative evaluation of six breakout groups by transcribing verbatim and reading the raw data iteratively. Transcripts were imported to the qualitative data analysis software MAXQDA 2022. Codes were developed inductively to organize the data, then we established links between the data and the study objectives, and built a framework of the underlying experiences from the participants. The final stage of data retrieval included defining codes and categories and triangulating findings by two different coders.

## Results

**Fig. 2:**
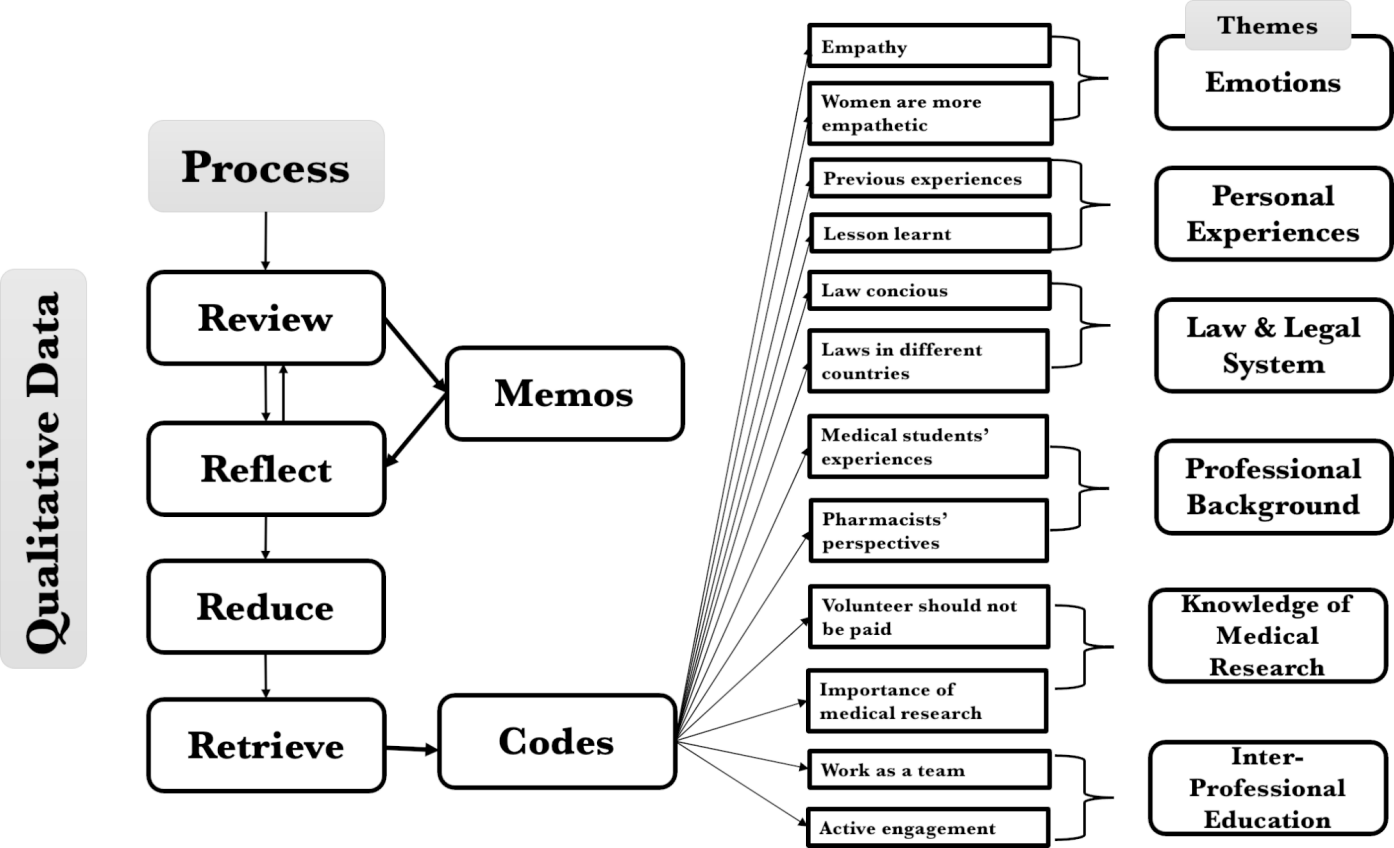
Qualitative evaluation process and emerging themes

Qualitative data derived from the recorded breakout group discussions about the ethical dilemmas and decisions on available options were presented according to six themes: (1) emotions, (2) personal experiences, (3) law and legal system, (4) professional background, (5) knowledge of medical research and (6) inter-professional education (Fig. 2).

### Emotions

One student from the group responded to case no.4 as “*If the patient cannot pay for himself, we can just channel our empathy and help them*” *(a student from group 4).* When reflecting upon the case of an adolescent male who presented with a suspicious sexually transmitted disease and requested not to share his medical findings with his parents, some students insisted to respect the adolescent’s wishes. A student from group 2 stated “*if the parents were informed about what’s going on, the child will be maybe abused or maybe punished”.* The student’s emotion-based approach was purely based on sympathy towards young patient who was struggling to face his parents with the news of a disease that he had acquired. However, other students responded “*I think laws and ethical should not be covered and interfering with some ‘empathy’, it should be pure law*” *(a student from group 4)* and *“Because he is minor, his parents should be notified*” *(a student from group 5)* when they recognized the individual’s emotion as a threat to the proper functioning of the legal system that could be imposed on the ethical issues in medical practice. It is important to note that the balance between emotions management and emotional intelligence may allow healthcare professionals to overcome emotional challenges in complex ethical situations. Although medical professionals may not be able to completely avoid emotional challenges, they can use effective stress management techniques and effective interpersonal skills to respond to these situations effectively [25].

Responding to the concerns about the issue of premarital screening and informing the partner about sexual transmitted diseases, interestingly, female students seemed to be more empathetic and concerned with the woman in the case scenario than male students and predominantly inclined to the option of either ‘refuse the patient’s wish and tell the future partner about the husband’s condition’ or ‘stop the procedure of issuing the certificate’. One student stated that “*I would tell the wife because the wife is also my patient, and I also think something contagious like this should be told*” *(a student from group 5)*. Another response to this issue included “*If he refuses to tell his wife, you would have to tell him that you cannot give him the premarital screening certificate*” *(a student from group 5)*. Overall, female students felt more inclined towards telling the woman about the diagnosis of her future partner in order to protect her future health and thus develop a sense of empathy towards her. Emotions play an important role in human decision-making and can be affected by external factors such as gender or culture [1].

### Personal experiences

Having past experience could assist medical practitioners in making difficult decisions based on uncertain and confusing information. A student recalled his personal experience “*Actually, I saw a very similar case like almost the exact same thing that’s happened in the hospital once recently…*” *(a student from group 4)*. Not only students’ personal experiences helped them in making decisions when experiencing ethical dilemmas, but also it could be one of the factors to consider for a patient. One student from group 5 commented to case no.1 “*what if the patient has had a bad experience before with being admitted involuntary to a psychiatric facility?*” *(a student from group 5)*.

### Law and legal system

As neither ethics nor laws stand alone, all of the presented cases in the ethics workshop included both ethical and legal components. When facilitators presented lists of alternative decisions on each specific ethical dilemmas, the students suggested options based on the legal system with several ethical issues in mind. The following are three quotes presented by the students across groups.

“*I think it depends on the country and the laws there. One of the reasons in some states in the US, actually they decided for sexually transmitted diseases not to inform the parents because the teenagers were not treated because they scared from their parents” (a student from group 6)*.

*“I think we should not give him a treatment because he’s under the age, and I think there is a law that we should take consent from his parents because he’s under 18” (a student from group 4)*.

*“I think there is a law that forces people to go into psychiatric care, even if they have a negative experience without their consent and the admission is mandatory as far as I know” (a student from group 5)*.

### Professional background

Healthcare professionals often take different approaches to decision-making based on their professional backgrounds. They might use alternative tactics, analyze various aspects of the ethical dilemmas, or assign different values to the options. When it came to the case of good medical practice prescribing antibiotics to the patient, a medical student commented “*Your job as a doctor, if the patient does not need it, then you don’t have to prescribe and it’s immoral. So why are you prescribing it?*” *(a student from group 2).* Also, this was the opinion echoed by other medical students while a pharmacy student shared her opinion as “*I talk as a pharmacist, they don’t like to move until they received antibiotics. Imagine this one. Yes, I need to dispense it, but maybe I can give him very mild one, let’s say, weak antibiotic*” *(a student from group 3)*.

### Knowledge of medical research

Commenting on the case of a clinical trial with healthy paid volunteers who would be infected with the COVID-19 virus using nasal drops, common views amongst students were that research participants should not be paid as volunteers would be harmed by exposure to the virus. There were some suggestions including  “*they paid the participants, so the participation is not for science, it’s for benefits for money*” and “*you might harm the patients, if they were healthy, they might have some serious consequences and complications from COVID-19” (a student from group 2)*. Whilst only a small number of participants shared their opinions for paying the volunteers in the advancement of medical research and one student stated “*I think because they’re paid volunteers, so obviously a written consent must be obtained, so they know that they would be exposed to the virus or they consented to it, so it’s justifiable*” *(a student from group 5)*. Another participant shared a thought on justification to conduct ethically accepted medical research “*Since this is a clinical trial and these patients have voluntarily accepted to be infected with the virus in order to assess for the efficacy of the side effects*” *(a student from group 6)*.

### Inter-professional education

Designing an ethics workshop with students from different medical colleges created an effective inter-professional learning activity, which is an important approach for preparing students in the healthcare field where collaboration and teamwork are essential skills. When the ethical dilemmas of prescribing antibiotics were discussed, students expressed their opinions and shared their agreement with other students *“I think we all agree that we would go with the second option, which is to provide the education and reassurance without prescribing” (a student from group 5)*. Moreover, students from various backgrounds and cultures learned in different ways as well as in different perspectives and it helped us to develop professional awareness and communication skills. For instance, when the same case was viewed by two different health profession students, their ethical decisions were different. A medical student said, *“As a doctor, I’m not going to do it, I would just educate the patient as much as I can”* while a pharmacy student stated that *“It’s like pressure on the pharmacists…I need to dispense it” (students from group 3)*. Presenting ethical dilemmas and discussing the alternative options to reach an optimum decision within a group with different professionals could assist students in their communication and confidence in their area of expertise and promote inter-professional teamwork which intern enhances the quality of patient care.

**Fig. 3:**
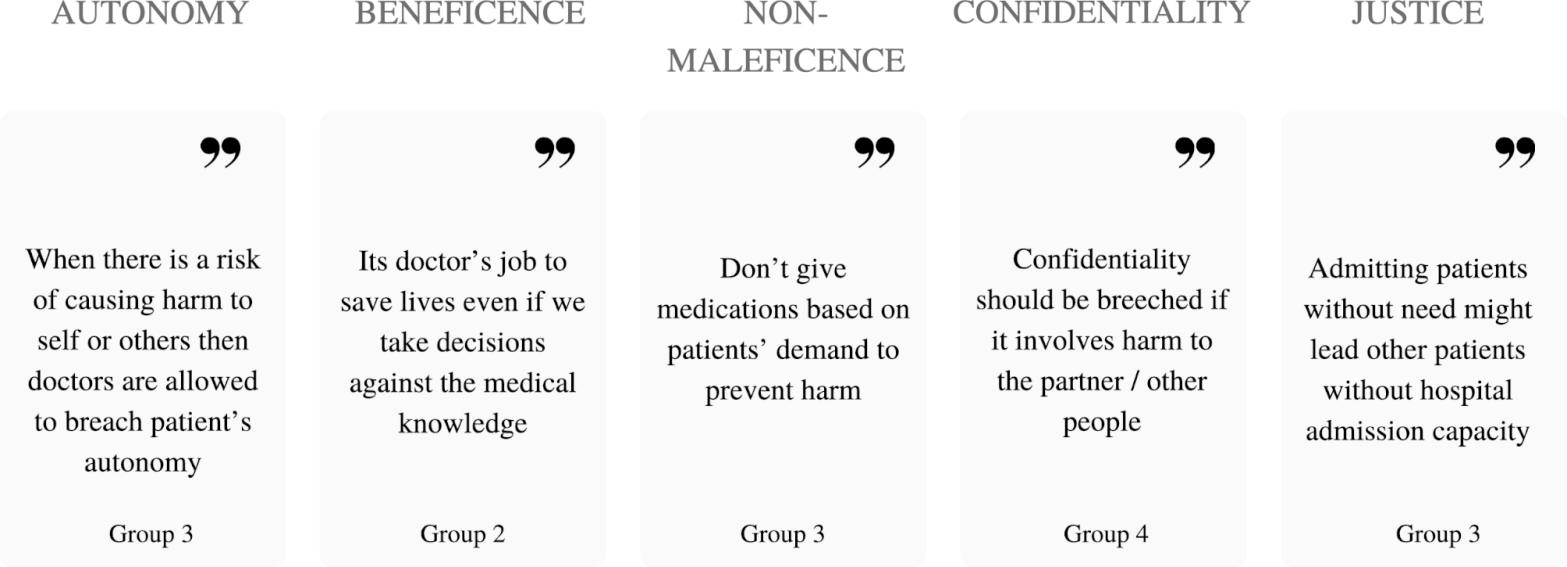
The application of ethical principles during group discussions.

**Fig. 4:**
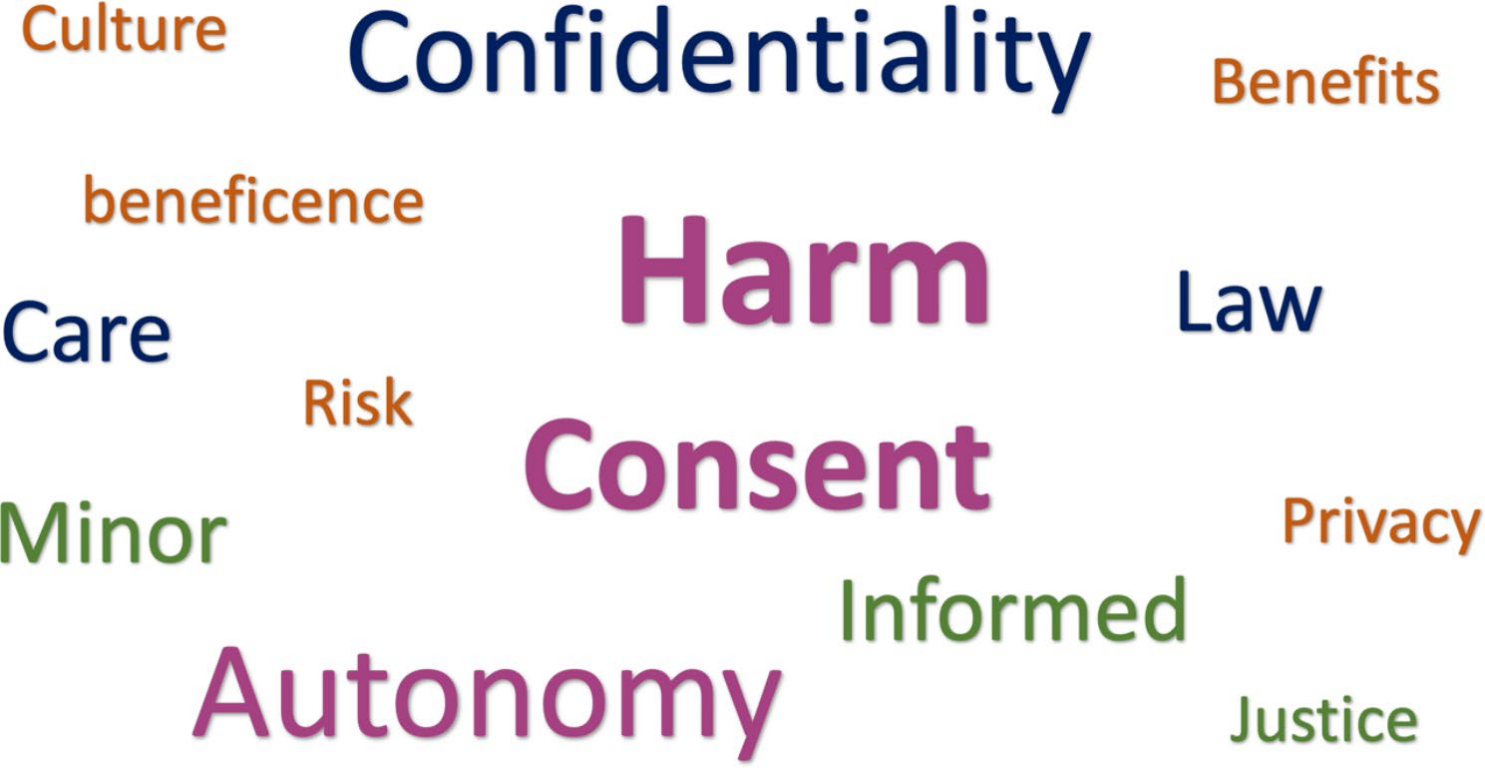
Word cloud of the most frequently used words during group discussions.

Mostl,y ethical dilemmas arise when ethical principles clash with each other. Students applied the learnt knowledge of ethical principles from the workshop and their previous experiences in their reasoning to reach ethical decisions. Some of the statements for the ethical principles applications in the group discussions are presented in the Fig. 3. The most discussed words in their reasoning process were autonomy, consent, harm, and confidentiality (Fig. 4).

## Discussion

Globally, despite having a plethora of published reports regarding ethical decision-making and factors influencing the ethical development in medical education [26, 27], to the best of our knowledge, there remains ambiguity about what makes the health professions students choose a solution over others which are also legally permissible and ethically defensible among ethical dilemmas. 

This study demonstrated six potential factors that could potentially influence the ethical decision-making by health professions students on ethical dilemmas.. These six potential influencing factors are emotions, personal experiences, law and legal systems, professional background, knowledge of medical research and inter-professional learning activity.

The first factor affecting ethical decisions is emotions. Health professionals are often unaware that they are being influenced by an unintentional emotional state. Thus, a transitory incidental emotion might influence their decisions on ethical dilemmas. Some students in our study disagreed with their fellows’ opinions about prioritizing emotions. Emotion is often viewed as an element of ethical decision-making that should not be considered since it interferes with logic. However, emotions play an essential role in moral decision-making by influencing the decision-making process and determining whether an individual acts out of principle or self-interest. This is supported by a study from the nursing field where researchers suggested that emotions should not be ignored as “irrational biases” to a rational ethical decision process but paying attention to one’s emotions could lead to better ethical decisions [28]. Empathy, a moral emotion surfaced mainly in the female participants in this study. Personal characteristics such as gender influence one’s priorities and values which are present in every work whether explicitly stated or not [29]. Add new Ref.here...Menezes P, Guraya SY, Guraya SS. A systematic review of educational interventions and their impact on empathy and compassion of undergraduate medical students. Frontiers in Medicine. 2021 Nov 8;8:758377. In our study, female students seemed to be more empathetic in the scenario where the rights of women were concerned. Although several reports have shown that women were more likely to make ethical choices than men in general, this study has found that women’s empathetic choices were situationally specific and this is supported by the previous observations in the study of Barnett and Karson [30].

Similar to emotions, justice is another intrinsic value and extrinsically it is a result of the application of the enforcement of the law. While some students prioritized emotions, others were conscious of the law and the legal system and propose solution based on that. Logical reasoning based on law and legal system was an obvious factor among factors influencing ethical decisions.

Personal experiences and professional background were other contributing factors for the students when they were asked to select the best possible option from a wide range of alternatives of ethical dilemmas. These influencing factors reflect a statement of the previous researcher’s work where he stated that “ethical decision is highly personal and often experiential” [25] Add new Ref here... Trede F, Macklin R, Bridges D. Professional identity development: a review of the higher education literature. Studies in higher education. 2012 May 1;37(3):365-84.

One of the presented case scenarios in the ethics workshop was about a clinical trial and students were encouraged to share the opinion on how to resolve the ethical dilemmas. Understanding medical research and its ethical implications in clinical trials such as compensation methods, favorable risk-benefit ratios, and informed consent could inform healthcare professionals’ or students’ perspectives on ethical dilemmas in medical research. The overarching purpose of clinical trials stems from the fact that a new management strategy or testing technique could be safely adopted in health care systems without harm to patients and society. However, this is a debatable issue as volunteers may be exposed to potential risks of a new drug or therapy which is being tested for the first time in clinical trials ADD new Ref here Doi SA, Furuya-Kanamori L, Xu C, Lin L, Chivese T, Thalib L. Controversy and debate: questionable utility of the relative risk in clinical research: paper 1: a call for change to practice. Journal of clinical epidemiology. 2022 Feb 1;142:271-9.. AND Herzberg J, Vollmer T, Fischer B, Becher H, Becker AK, Honarpisheh H, Guraya SY, Strate T, Knabbe C. SARS-CoV-2-antibody response in health care workers after vaccination or natural infection in a longitudinal observational study. Vaccine. 2022 Jan 21;40(2):206-12. 

As the ethics workshop was designed is an inter-professional learning climate, group discussions were carried out among students from different health sciences colleges. This interprofessional atmosphere influenced the way students conversed and compared their experiences between different contexts. Therefore this study proposed that interprofessional education and collaboration is a convenient vehicle for medical students to learn the core principles of medical ethics. other reports have also argued the substantial role of interprofessional education in the medical field Add new Ref...Sulaiman N, Rishmawy Y, Hussein A, Saber-Ayad M, Alzubaidi H, Al Kawas S, Hasan H, Guraya SY. A mixed methods approach to determine the climate of interprofessional education among medical and health sciences students. BMC medical education. 2021 Dec;21:1-3. In our study, students were able to communicate their ethical judgements in simple yet effective ways that reflected the four ethical principles; autonomy, beneficence, non-maleficence, and justice. The word harm was the most common word used during small group interactive discussions. Students justified their choices on ethical dilemmas which based on either weighing between greater harm and lesser harm or preventing harm altogether.

The scope of our study was limited in terms of the qualitative data, as the study only included recorded video data and did not include other effective qualitative data collection methods (e.g., in-depth interviews and focus group discussion). Notwithstanding, despite the relatively limited qualitative data, this work was able to shed new light on ethical development in medical education by gaining students’ perspectives in dealing with complex clinical scenarios.

## Conclusion

The study has identified six potential influencing factors for the real practice based clinical scenarios where ethical dilemmas were presented and ethical principles were clashing with each other. The findings of our study explained how health professions students resolve ethical dilemmas in their ethical reasoning process. These findings could provide valuable information for the faculty to develop various strategies to assist students in resolving various ethical dilemmas. Additionally, these valuable insights could contribute to the development of teaching methods to facilitate the resolution of ethical dilemmas for health profession students. However, considerably more qualitative inquiries are essential to determine the contributing factors to transform health professions students into ethically sound clinical leaders.

## Data Availability

The datasets used and/or analyzed during the current study available from the corresponding author, Prof Nabil Sulaiman ( nsulaiman@sharjah.ac.ae) on reasonable request.

## List of abbreviations

CMHS: College of Medicine and Health Sciences
CoM: College of Medicine
CDM: College of Dental Medicine
CoP: College of Pharmacy
UoS: University of Sharjah
UAE: United Arab Emirates
UAEU: United Arab Emirates University
